# Reply to Aldè et al. Comment on “Manchaiah et al. Social Representations of “Tinnitus” and “Health” among Individuals with Tinnitus Seeking Online Psychological Interventions. *Audiol. Res.* 2023, *13*, 207–220”

**DOI:** 10.3390/audiolres13040057

**Published:** 2023-08-14

**Authors:** Vinaya Manchaiah, Pierre Ratinaud, Eldre W. Beukes

**Affiliations:** 1 Department of Otolaryngology—Head and Neck Surgery, University of Colorado School of Medicine, Aurora, CO 80045, USA; 2 UCHealth Hearing and Balance, University of Colorado Hospital, Aurora, CO 80045, USA; 3 Virtual Hearing Lab, Collaborative Initiative between University of Colorado School of Medicine and University of Pretoria, Aurora, CO 80045, USA; 4 Department of Speech-Language Pathology and Audiology, University of Pretoria, Pretoria 0002, Gauteng, South Africa; 5 Department of Speech and Hearing, Manipal College of Health Professions, Manipal Academy of Higher Education, Manipal 576104, Karnataka, India; 6 Laboratoire d’Études et de Recherches Appliquées en Sciences Sociales (LERASS), University of Toulouse, 31000 Toulouse, France; pierre.ratinaud@univ-tlse2.fr; 7 Vision and Hearing Sciences Research Group, School of Psychology and Sport Science, Anglia Ruskin University, Cambridge CB1 1PT, UK; eldre.beukes@aru.ac.uk

We would like to thank Dr. Aldè and his colleuage’s for their thoughtful comments [1] regarding our recent publication “Social representations of “tinnitus” and “health” among individuals with tinnitus seeking online psychological interventions” [2]. 

Dr. Aldè and colleagues’ comment about the relatively small sample size in the study (n = 399) for a social representation study is valid. However, there is no statistical methodology for estimating the sample size for such studies. Also, considering the exploratory nature of the published study, we think that the sample size was reasonable. However, we fully agree that repeating the study with a larger sample size (n > 1000) as well as with appropriate sampling method would yield results that are more generalizable.

The study was secondary analysis of data gathered during clinical trials [3,4,5]. For this reason, we only included participants who were seeking online psychological interventions. However, as acknowledged in our study limitations, this population may not be a good representation of the clinical population that we see as well as all those who experience tinnitus. For this reason, the study results must be viewed with caution. We also agree that future research on this topic should be performed on clinical sample. 

The study was conducted completely online without any in-person consultation during the COVID-19 pandemic. For this reason, we do not have details on participants clinical variables such as hearing status (pure-tone audiometry results), hearing device use and other medical conditions. To ensure that the study participants did not have huge burden of completing online questionnaires, we kept the questionnaire data collection limited to key demographics and outcome measures necessary for the clinical trials. We have discussed the participants characters based on these in the social representation study [2] as well as on our clinical trials [3,4,5]. However, we fully agree that having a complete picture of participants clinical and health variables could have provided much better understanding. 

We highly appreciate Dr. Aldè and his colleague’s thoughtful comments on this manuscript. We will keep these comments in mind when designing the future studies in this area of study.
